# A systematic review of observational studies on the association between diet quality patterns and visceral adipose tissue

**DOI:** 10.1017/S000711452400179X

**Published:** 2024-12-14

**Authors:** Annalena Thimm, Gertraud Maskarinec, Cherie Guillermo, Katharina Nimptsch, Tobias Pischon

**Affiliations:** 1 Max Delbrück Centrum für Molekulare Medizin, Berlin, Germany; 2 University of Hawaii Cancer Center, Honolulu, HI, USA

**Keywords:** Body fat composition, Diet quality, Obesity, Dietary patterns

## Abstract

Beyond obesity, visceral adipose tissue (VAT) has emerged as an important predictor of chronic disease, but the role of diet quality patterns (DQP) in VAT development is not well defined. Therefore, we conducted a systematic review of how various DQP are associated with VAT via literature searches in PubMed and EMBASE. We included observational investigations in disease-free adults/adolescents that related DQP to VAT assessed by imaging methods. The studies were evaluated separately for *a priori* and *a posteriori* DQP and according to design differences. Study quality was assessed using the Risk of Bias in Non-randomised Studies of Interventions tool. Of the 1807 screened articles, thirty-five studies met the inclusion criteria. The majority of *a priori* indices, for example, the Healthy Eating Index, showed significant inverse associations with VAT, while only a small proportion of *a posteriori* patterns were related to VAT. Results did not differ substantially by the method of exposure and outcome assessment or between studies with (*n* 20) or without (*n* 15) body-size adjustment, but significant findings were more common in younger *v*. older individuals, USA *v*. other populations and investigations with moderate *v*. serious risk of bias. The heterogeneity of the existing literature limited the ability to quantify the magnitude of the associations across studies. These findings suggest that a high-quality diet, as assessed by *a priori* DQP, is generally inversely associated with VAT, but results for *a posteriori* DQP are less consistent. As associations persisted after adjusting for body size, diet quality may beneficially influence VAT beyond its association with obesity.

As obesity prevalence has risen around the world, research on dietary factors influencing BMI has proliferated^(1)^. Of particular interest is the distribution of body fat, foremost the amount of visceral (VAT) *v*. subcutaneous adipose tissue, an important predictor of chronic disease beyond BMI^(2,3)^. VAT adipocytes are more metabolically active and insulin-resistant than those in subcutaneous adipose tissue, which may be among the mechanisms responsible for the higher incidence of type 2 diabetes^(4)^, postmenopausal breast cancer^(5)^ and colorectal cancer^(6)^ associated with abdominal adiposity^(7–9)^. Given that waist circumference cannot distinguish between subcutaneous adipose tissue and VAT^(10)^, imaging techniques, that is, computed tomography (CT), MRI, dual-energy X-ray absorptiometry (DXA) or ultrasound need to be applied for their assessment^(2)^.

Dietary patterns as a method of assessing overall dietary intake instead of individual food consumption have emerged as a useful approach to assess overall diet quality^(11)^, which can be classified into *a priori* and *a posteriori* diet quality patterns (DQP). *A priori* DQP, also known as diet quality indices, are based on dietary recommendations and scientific evidence about diet and health-related outcomes^(12)^. They evaluate adherence to specific dietary guidelines, such as the Healthy Eating Index (HEI)-2010^(13,14)^, the Alternate HEI^(15)^, the Alternate Mediterranean Diet Score^(16)^, the Dietary Approaches to Stop Hypertension^(17)^, the Dietary Inflammatory Index^(18)^ and several lesser-known ones^(19–21)^. In contrast, *a posteriori* DQP are identified through exploratory data-driven approaches^(11)^, for example, factor analysis, principal component analysis or cluster analysis^(22)^, which use observed correlations among dietary variables to create a pattern based on reported intakes and allow the discovery of new diet-disease associations^(23)^. Despite differences in recommended food groups across indices, high-quality diets generally score high in fruits, vegetables, whole grains, dairy products, nuts and legumes, whereas meat products, processed foods, refined grains, Na and sugar-sweetened beverages lower the scores^(24)^.

Early on, a relation of a high Mediterranean diet with lower waist circumference as a measure of abdominal fat was described in a European study^(25)^. Starting 20 years ago, MRI and CT-based imaging studies reported significant inverse associations of DQP with VAT^(26,27)^ with approximately 15 % lower values in the highest *v*. lowest tertile^(28,29)^. Given the rising number of reports exploring the association between DQP and VAT as new imaging methods were developed, it is important to understand the current state of knowledge. Therefore, the aims of the present project were to summarise findings for the association of *a priori* and *a posteriori* DQP with VAT assessed by an imaging technique and to evaluate differences according to design features, that is, the type of DQP, dietary assessment method, imaging technique, age, bias, location and adjustment for BMI or an alternate measure of body size.

## Methods

### Overview

This systematic review was carried out in accordance with the Preferred Reporting Items for Systematic Reviews and Meta-Analyses (PRISMA) 2020 statement^(30)^. The aim was operationalised according to the PI/ECO (Population, Intervention/Exposure, Comparison, Outcome) scheme after the inclusion and exclusion criteria had been determined *a priori*. We included studies conducted in disease-free children/adolescents or adults recruited from a general population, that is, not selected as patients with an underlying condition, using *a priori* or *a posteriori* DQP as exposure and VAT as outcome.

### Search strategy and study selection

Based on pre-defined eligibility criteria, synonymous keywords for each search term were included in the search string (online Supplementary Table 1) after registering the protocol on PROSPERO (CRD42022366565). The systematic search was conducted on 22 October 2022 in PubMed and EMBASE via OVID using the same search string. The 628 hits on PubMed and 958 hits on EMBASE with 573 duplicates were imported into the CAMARADES Preclinical Systematic Review and Meta-analysis Facility for title–abstract screening (https://app.syrf.org.uk/) last accessed on 27 December 2022. To update the literature, a new search using the same strategy was performed in January 2024, which identified seventy-nine additional hits on PubMed and 142 on EMBASE with seventy-one duplicates.

**Fig. 1.**
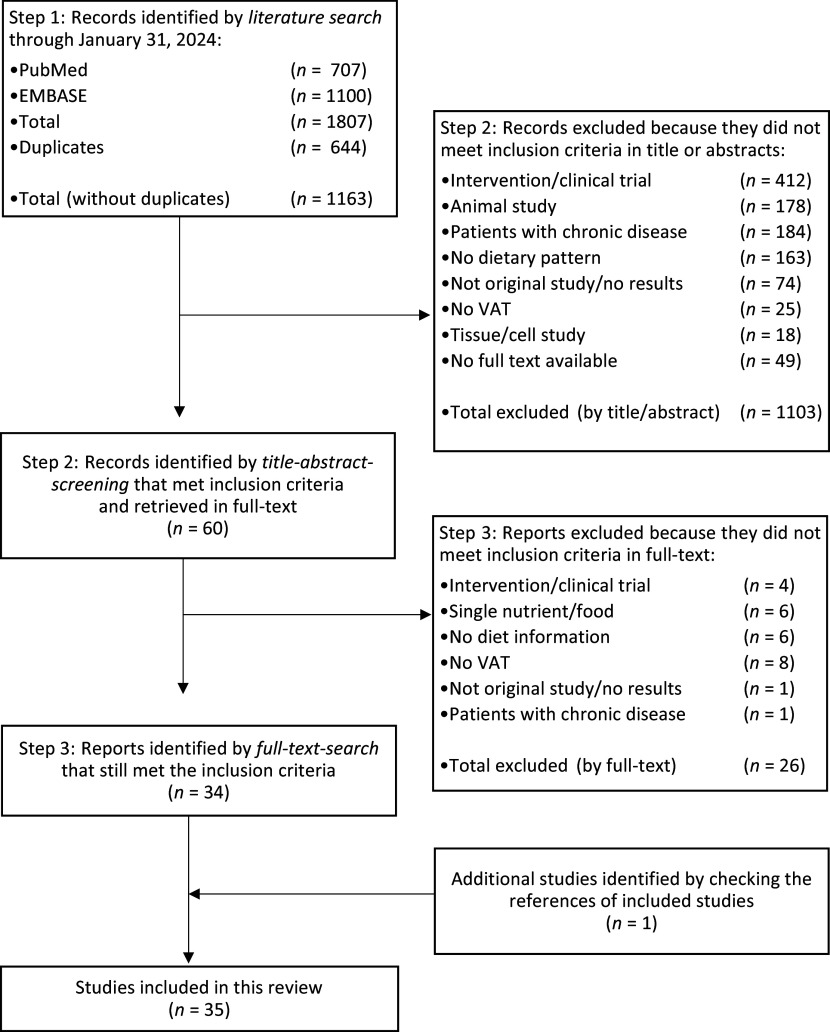
Flow chart of literature search and study selection.

As shown in the PRISMA flow diagram (Fig. 1), this review was limited to human observational studies written in the English language, which included adults or children older than 5 years. Studies with patients or pregnant women were excluded. Diet quality had to be assessed by *a priori* or *a posteriori* DQP. The original studies needed to allow a quantitative comparison of VAT, measured by one of four established imaging methods (MRI, CT, DXA or ultrasound)^(2)^. CT uses X-rays to produce cross-sectional images of the body and differentiate between fat and other tissues, while MRI distinguishes tissue types based on their water and fat content in the abdominal region where VAT is concentrated^(31)^. In addition, a DXA method, which has been repeatedly validated against CT and MRI^(32–34)^, analyses the differential attenuation of two X-ray beams with different energy levels to assess the amount of fat tissue and to estimate VAT based on fat distribution patterns through specific algorithms^(31)^. Ultrasound has been applied less commonly so far; body fat is measured 1 cm above the umbilicus as the distance between the anterior wall of the aorta and the posterior surface of the *rectus abdominis* muscle at the level of linea alba using an ultrasonographic probe^(27,35,36)^.

Abstracts without a published full-text manuscript were excluded. The title–abstract screening on Systematic Review and Meta-analysis Facility was performed independently by two reviewers (AT and GM) to ensure that all relevant titles were retrieved after removing duplicates using a reference database. Each reviewer assigned a reason for exclusion, that is, animal study, intervention/randomised controlled trial, not an original study, for example, review/no original data, tissue/cell study, patients with chronic disease, no VAT measure or no DQP, and disagreements were solved by a third reviewer (KN). For the remaining papers, the full texts were screened independently by the two reviewers, and the third reviewer resolved conflicts. The references of all studies were screened to identify eligible reports to be added.

### Data extraction

For studies that fulfilled the inclusion criteria, detailed information relevant to our research question was extracted: first author, year of publication, country, study design, sample size, ethnicity, mean age, sex, diet assessment method, DQP approach (*a priori* or *a posteriori*) and type, mean VAT value and VAT assessment method, for example, MRI, DXA, CT or ultrasound. For *a posteriori* DQP, we recorded the statistical technique, for example, principal component analysis, plus the number of food or nutrient items included. We summarised statistical methods, covariates, comparison groups (categorical or continuous), a unit of VAT measure and effect measures for VAT, that is, adjusted means, 95 % CI, standard error, *P*-value and type (e.g. tertiles or continuous DQP). If multiple models were applied, the adjusted mean for the model controlling for body size, that is, BMI, weight and total body fat, was extracted. If none of the models controlled for body size, the results for the model with the most comprehensive adjustment set were extracted. In studies reporting no exact mean values for age and sex, an approximate mean value was estimated. After finishing the extraction, the second reviewer checked the information.

### Assessment of quality and risk of bias

With the help of the Risk of Bias in Non-randomised Studies of Interventions (ROBINS-I) tool^(37)^, the two reviewers independently assessed the methodological quality of the included studies (online Supplementary Table 2). This Cochrane assessment tool, when adapted for observational studies, consists of six bias domains: (1) confounding, (2) selection of participants, (3) exposure assessment, (4) missing data, (5) measurement of the outcome and (6) selective reporting of the results. The overall judgement of risk of bias was categorised as low, moderate or serious^(37)^. If at least one domain was rated as serious, then the overall judgement was serious. If a low or moderate risk of bias was found for all domains, then the overall judgement was classified as moderate.

### Analysis

Tables showing the VAT-effect measures sorted by DQP and a graphical representation of the direction of the observed association were created from the data extraction tables. Also, a summary table presents the study results by design characteristics. As these varied across studies, a separate narrative synthesis was prepared for each DQP. VAT-effect measures were compared between high and low DQP scores either in quantiles or as continuous measures. When available, results adjusted for body size were selected to demonstrate the association of DQP with VAT beyond the influence of BMI.

## Results

From a total of 1807 hits in both databases (Fig. 1), 644 duplicates were removed. Of the remaining 1163 publications, 1103 were excluded due to the following reasons (Fig. 1): interventions/clinical trials (*n* 412), animal studies (*n* 178), study population consisted of patients (*n* 184), no DQP (*n* 163) and other criteria (*n* 166). After screening the remaining sixty publications in full text (see the ‘Methods’ section), thirty-four publications plus one study found in the bibliography of a selected paper were included, thus, resulting in thirty-five population-based observational studies for this review.

Of the thirty-five publications, twenty-six examined *a priori* (Table 1) and nine *a posteriori* DQP (Table 2). All had a cross-sectional design and, with the exception of six primary analyses^(27,29,35,39,47,58,65)^, were secondary analyses of prospective cohorts. The analytical sample sizes ranged from 59^(39)^ to 9640^(48)^ participants. Two studies included only women^(62,63)^, one included only men^(39)^ and three were performed on adolescents (one in Hispanic freshmen 18–19 years, one in Brazilian students 10–16 years and one in low-income US kids 10–16 years)^(44,47,58)^, but no studies in children were identified. As to adults, four studies reported a mean age in the 20s, two in the 30s and five in the 40s, whereas the remaining ones were conducted in populations 50 years and older with twelve studies in the 50s and nine in the 60s. The majority of studies reported mean BMI in a range of 25–30 kg/m^2^; six studies showed values of 30 kg/m^2^ or higher and eight studies a mean below 25 kg/m^2^. As to location, sixteen were conducted in the USA^(21,26,28,29,38,43–45,47,48,50–53,55,56)^, four in South Africa^(54,62–64)^ and three in Germany^(46,59,60)^, while the remaining came from Italy^(27,35)^, Spain^(40,42)^, Brazil^(39,58)^, the Netherlands^(20)^, Portugal^(49)^, Sweden^(41)^, Japan^(61)^, Korea^(57)^ or China^(65)^. Twelve studies investigated multiple ethnic groups^(29,38,43–45,48,50–52,55,56)^, while the remaining studies focused on one ethnic group (*n* 18) or did not report any details (*n* 5). Dietary assessment tools included different types of FFQ (*n* 26), but short questionnaires (*n* 4), 24-h recalls (*n* 3), a 3-d food record (*n* 1) and a diet history (*n* 1) were also used. VAT was measured by DXA (*n* 14), MRI (*n* 13), CT (*n* 6) or ultrasound (*n* 2).

**Table 1.** Characteristics of studies using *a priori* diet quality patterns

Author year	Country*	*N*	Ethnicity†	% Female	Age (years)‡		BMI (kg/m^2^)‡		DQP§	Diet assessment||	VAT¶
					Mean or median	Sd or IQR	Mean or median	Sd or IQR			
Bellissimo 2020^(38)^	USA	693	W, A, AA, AI	65 %	49	12	28·1	0·3	AHEI, DASH, MED	Block FFQ	DXA
Bertoli 2015^(27)^	IT	4388	W	73 %	46	37–56	27·9	25·0–31·0	MED	14-item Qx	Ultrasound
Corrêa 2022^(39)^	BR	59	W, AA, O	M only	26	4	25·4	4·5	E-DII	3-d records	DXA
De Amicis 2020^(35)^	IT	416	W	70 %	50	13	29·6	6·3	MED	14-item Qx	Ultrasound
Flor-Alemany 2020^(40)^	ES	176	N/A	100 %	53	4	≥25:61 %		MED	FFQ (173 items)	DXA
Fridén 2022^(41)^	SE	286	N/A	53 %	50		25·8	23·4–28·6	UPF	sQFFQ (140 items)	MRI
Galmes-Panades 2018^(42)^	ES	1231	W	47 %	65	5	32·5	3·3	MED	FFQ (17 items)	DXA
Hennein 2019^(28)^	USA	1677	N/A	50 %	51	10	27·5	5·0	MED	FFQ (126 items)	CT
Holthaus 2023^(43)^	USA	163	A, AA, AI, W, O	58 %	34	6	30·4	6·6	MIND, MED, DASH, HEI	FFQ (124 items)	DXA
Hu 2023^(44)^	USA	192	AA, W, O	52 %	13	2	71·3 ± 30·2 (percentile)		HEI	24-h recalls (3)	MRI
Isanejad 2023^(45)^	USA	3017	AA, W	56 %	25	0·1	24·4	0·19	HEI	FFQ (46 items)	CT
Jennings 2024^(46)^	DE	620	W	41 %	61	12	27·2	4·4	aMED	FFQ (112 items)	MRI
Landry 2019^(47)^	USA	92	H	51 %	19	0·5	23·8	3·9	HEI	24-h recalls (3–4)	MRI
Liu 2023^(48)^	USA	9640	AA, A, H, W, O	49 %	39	10	NA		UPF	24-h recalls (1–2)	DXA
Lopes 2023^(49)^	PT	70	W	74 %	23	7	22·0	2·8	MED	14-item Qx	DXA
Lozano 2022^(50)^	USA	1655	A, AA, H, NH, W	33 %	69	3	28·0	4·8	E-DII	QFFQ (180 items)	MRI
Maskarinec 2017^(51)^	USA	1861	A, AA, H, NH, W	50 %	69	3	28·0	4·8	HEI, AHEI, aMED, DASH	QFFQ (180 items)	MRI
Maskarinec 2020^(52)^	USA	1861	A, AA, H, NH, W	50 %	69	3	28·0	4·8	HEI	QFFQ (180 items)	MRI
Molenaar 2009^(53)^	USA	925	W	49 %	50	10	F: 27·0, M: 28·3		DGAI	sQFFQ (112 items)	CT
Mtintsilana 2019^(54)^	ZA	190	AA	F only	53	48–59	33·5	29·8–38·8	E-DII	FFQ	DXA
Odegaard 2022^(55)^	USA	3156	AA, W	57 %	26	23–29	23·1	21·1–25·8	Fast-food	Diet history	CT
Panizza 2020^(29)^	USA	540	A, AA, H, NH, W	56 %	46	17	27·2	7·8	HEI	sQFFQ (134 items)	DXA
Ratjen 2020^(21)^	USA	578	N/A	43 %	62	55–71	27	24–29	PDI, hPDI, uPDI	sQFFQ (112 items)	MRI
Shah 2016^(56)^	USA	5079	W, AA, A, H	53 %	62	53–70	27·8	25–31·3	Diet Quality Score	sQFFQ (127 items)	CT
Shim 2023^(57)^	KR	2303	A	62 %	52	9	24·2	3·0	UPF, HEI	FFQ (112 item)	DXA
Van Eekelen 2018^(20)^	NL	258	N/A	57 %	56	6	26·2	4·4	DHD	FFQ (125 items)	MRI

* *ISO* 3166–1 *alpha-2 codes*.

† A, Asian; AA, African (American); AI, American Indian/Alaska Native; H, Hispanic; NH, Native Hawaiian; O, Other; W, white.

‡ Mean ± sd or median (IQR).

§ AHEI, Alternate HEI; aMED, Alternate Mediterranean Diet Score; DASH, Dietary Approach to Stop Hypertension; DGAI, Dietary Guidelines Adherence Index; DHD, Dutch Dietary Guidelines Index; DQP, diet quality pattern; E-DII, Energy-Adjusted Dietary Inflammatory Index; hPDI, healthy plant-based diet index; MED, Mediterranean Diet Score; MIND, Mediterranean Intervention for Neurodegenerative Delay; PDI, plant-based diet index; UPF, ultra-processed food; uPDI, unhealthy plant-based diet index.

|| Qx, questionnaire; (s)QFFQ, (semi-)quantitative FFQ.

¶ CT, computed tomography; DXA, dual-energy X-ray absorptiometry; VAT, visceral adipose tissue.

**Table 2.** Characteristics of studies using *a posteriori* diet quality patterns

Author year	Country*	*N*	Ethnicity†	% Female	Age (years)‡		BMI (kg/m^2^)‡		DQP, *N* food items *N* patterns§	Diet assessment||	VAT¶
					Mean or median	Sd or IQR	Mean or median	Sd or IQR			
De Paula Mancilha 2022^(58)^	BR	231	H	46 %	13	1	F: 20·9 M: 21·2	0·37 0·4	PCA, 12 food items 2 patterns	FFQ (12 items)	DXA
Di Giuseppe 2019^(59)^	DE	553	W	45 %	61	60–62	27·5	27·1–27·8	RRR, 39 food groups One score	sQFFQ (112 items)	MRI
Fischer 2015^(60)^	DE	583	W	41 %	61	12	27·2	4·4	PCA and PLS, 87 energy-adjusted nutrient-groups 11 patterns	FFQ (112 items)	MRI
Ito 2019^(61)^	JP	829	A	36 %	52	8	M: 23·5 F: 21·4	2·9 3·1	PCA, energy-adjusted 52 food and beverage items 2 patterns	BDHQ (46 items)	MRI
Liu 2013^(26)^	USA	1775	AA	61 %	47	10	30·8	0·3	PCA, 31 food groups 3 patterns	FFQ (158 items)	CT
Makura-Kankwende 2020^(62)^	ZA	498	AA	100 %	49	45–53	33·7	28·1–39·0	PCA, energy-adjusted 25 nutrients 3 patterns	QFFQ (214 items)	DXA
Makura-Kankwende 2022^(63)^	ZA	132	AA	100 %	53	50–58	33·7	28·1–39·2	PCA, energy-adjusted 25 nutrients 3 patterns	QFFQ	DXA
Ratshikombo 2021^(64)^	ZA	760	AA	46 %	54	6	M: 25·5 F: 33·2	5·9 6·5	PCA, 25 nutrients 4 patterns	QFFQ	DXA
Yin 2020^(65)^	CN	1432	A	60 %	57	7	M: 24·1 F: 23	2·9 3	PCA, energy-adjusted 21 food groups 4 patterns	FFQ (81 items)	MRI

* *ISO* 3166–1 *alpha-2 codes*.

† A, Asian; AA, African (American); H, Hispanic; O, Other; W, white.

‡ Mean ± s d or median (IQR).

§ PCA, principal component analysis; PLS, partial least squares regression; RRR, reduced-rank regression.

|| BDHQ, brief-type self-administered diet history questionnaire; (s)QFFQ, (semi-)quantitative FFQ.

¶ CT, computed tomography; DXA, dual-energy X-ray absorptiometry; VAT, visceral adipose tissue.

*A priori* DQP (Table 1) included Mediterranean Diet Score (MED) (*n* 10), several versions of the HEI (*n* 8), Alternate HEI (*n* 2), Dietary Approaches to Stop Hypertension (*n* 3) and one each of the Dietary Guidelines for Americans Adherence Index, Dutch Dietary Guidelines Index, plant-based diet index, Diet Quality Score and fast-food intake. The Energy-Adjusted Dietary Inflammatory Index (E-DII) (*n* 3) and the ultra-processed food (UPF) according to NOVA classification (*n* 3) were scored reversely; that is, a lower score represents better diet quality (Table 1). *A posteriori* DQP (Table 2) were mostly identified through principal component analysis with the exception of one study each using partial least squares regression or reduced-rank regression^(59)^. As expected, they usually derived specific patterns with different labels.

The results of all thirty-five investigations, classified as no, partial (mixed findings for *a posteriori* DQP) or significant association, that is, higher diet quality associated with lower VAT, indicated several differences according to design characteristics (Table 3). Of studies investigating *a priori* DQP, 81 % showed significant associations in the expected direction, but the findings for *a posteriori* DQP were more likely to be null. By geographic location, the proportion of significant results was higher for US studies than in other areas. By age, more significant findings were seen in younger than older participants (79 *v*. 52 %). Investigations in multi-ethnic populations were more likely to indicate significant associations than studies with one group only (82 *v*. 44 %). No major differences were seen by dietary assessment method, imaging method, year of publication and body-size adjustment. Studies with and without body-size adjustment showed a similar rate of significant findings (65 *v*. 60 %).

**Table 3.** Summary of included studies according to design characteristics

Characteristics	Category	Association between DQP and VAT* (%)						Total
		None		Partial		Significant‡		
		*n*	%	*n*	%	*n*	%	
All studies		5	14	8	13	22	63	35
Diet quality pattern	*A priori*	5	19	0	0	21	81	26
	*A posteriori*	0	0	8	89	1	11	9
Dietary assessment	FFQ	4	16	6	24	15	60	25
	Other	1	10	2	20	7	70	10
Imaging method†	MRI	2	14	3	21	9	64	14
	CT	1	17	1	17	4	66	6
	DXA	1	8	4	31	8	61	13
	Ultrasound	1	50	0	0	1	50	2
Age range	<50 years	0	0	3	21	11	79	14
	≥50 years	5	24	5	24	11	52	21
Ethnic composition	One group	3	17	7	39	8	44	18
	≥2 groups	0	0	1	8	11	92	12
	No information	2	40	0	0	3	60	5
Year of publication	<2020	1	8	3	25	8	67	12
	≥2020	4	17	5	22	14	61	23
Geographic location	USA	1	6	1	6	14	88	16
	Europe	3	30	1	1	6	60	10
	Africa	0	0	3	75	1	25	4
	Asia	1	33	2	67	0	0	3
	S. America	0	0	1	50	1	50	2
Body size adjustment	Yes	3	15	4	20	13	65	20
	No	2	13	4	27	9	60	15
Overall risk of bias	Strong	4	14	8	29	16	57	28
	Moderate	1	14	0	0	6	86	7

* DQP, diet quality pattern; VAT, visceral adipose tissue.

† CT, computed tomography; DXA, dual-energy X-ray absorptiometry.

‡ Significant association of higher diet quality with lower VAT.

Looking at the HEI and similar DQP (Table 4), all reported relations were significantly inverse. For example, of the seven studies investigating the HEI^(29,43–45,51,52)^, the HEI-2015 was inversely associated with the Coronary Artery Risk Development in Young Adults (CARDIA) study with respective VAT values of 137 *v*. 117 cm^3^ in the lowest *v*. highest quantile^(45)^ as well as in the Multiethnic Cohort study with lower median VAT values (161 *v*. 175 cm^2^) in the highest than the lowest tertile^(52)^. Among Hispanic adolescents, a one-point increase in HEI-score was associated with a 1·49 ml lower VAT^(47)^. The Alternate HEI also demonstrated a significant inverse association with VAT^(38,51)^ as did the Dietary Guidelines for Americans Adherence Index in a study not adjusted for body size^(53)^ and the Dutch Dietary Guidelines Index after adjustment for body size^(20)^.

**Table 4.** Relation of *a priori* diet quality patterns with visceral adipose tissue (VAT) – part 1

Author year	DQP*	Baseline VAT†	VAT-effect measures by DQP‡	*P*	Body size adjusted	Association with VAT§
Landry 2019^(47)^	HEI	NA	C: −1·49 (–3·13, 0·15) ml	0·001	Y	↓
Panizza 2020^(29)^	HEI	481 ± 305 cm^2^	T1: 529 (495, 563) cm^3^ T3: 455 (419, 492) cm^3^	0·001	Y	↓
Maskarinec 2020^(52)^	HEI F	135 ± 62 cm^2^	T1: 144 (138, 149) cm^2^ T3: 123 (118, 128) cm^2^	<0·0001	Y	↓
	HEI M	202 ± 90 cm^2^ (clinic visit)	T1: 206 (198, 212) cm^2^ T3: 193 (186, 201) cm^2^	0·02	Y	↓
Isanejad 2023^(45)^	HEI	NA	QT1: 137 (134, 140) cm^3^ QT5: 117 (114, 120) cm^3^	<0·001	Y	↓
Holthaus 2023^(43)^	HEI	608 ± 324 g	C: −0·22 g	<0·01	N	↓
Hu 2023^(44)^	HEI	0·55 ± 0·47 kg	T1: 0·69 ± 0·03 kg T3: 0·57 ± 0·03 kg	0·01	Y	↓
Maskarinec 2017^(51)^	HEI	481 ± 305 cm^2^	T1: 529 (495, 563) cm^3^ T3: 455 (419, 492) cm^3^	NA	Y	↓
	AHEI	168 ± 84 cm^2^	T1: 172 (167, 177) cm^2^ T3: 163 (159, 165) cm^2^	NA	Y	↓
Bellissimo 2020^(38)^	AHEI	0·88 kg	C: –0·02 ± 0·003 kg (±se)	<0·001	N	↓
Molenaar 2009^(53)^	DGAI F	1308 ± 813 cm^3^	C: −139 (–222, −57) cm^3^	0·001	N	↓
	DGAI M	2160 ± 969 cm^3^	C: −243 (–353, −132) cm^3^	<0·0001	N	↓
Van Eekelen 2020^(20)^	DHD	NA	C: −2·3 (–3·5, −1·0) cm^2^	NA	Y	↓
	DHD F	T1–T3: 70, 68, 63 cm^2^	C: −1·4 (–2·8, −0·1) cm^2^	NA	Y	↓
	DHD M	T1–T3: 124, 115, 104 cm^2^	C: −2·3 (–4·4, −0·2) cm^2^	NA	Y	↓

* AHEI, Alternate HEI; DGAI, Dietary Guidelines Adherence Index; DHD, Dutch dietary Guidelines Index; HEI, Healthy Eating Index.

† Mean ± sd or median (IQR).

‡ C, continuous score, reported as ß (95 % CI) unless specified values; T, tertile; QT, quintile.

§ ↓, significant inverse association.

Of the eight studies investigating the original MED (Table 5), only two studies adjusted for body size; one found significant 50 cm^3^ less VAT accumulation for each 1-sd higher MED score^(28)^, but the other one reported null findings^(35)^. Of the remaining six studies, four studies described an inverse association with VAT with significant lower values of –0·05 cm^3(27)^, –0·06 kg^(38)^ and –0·19 g^(43)^ per 1 unit MED score and a significant difference between a high *v*. low score with VAT values (106 *v*. 267 cm^3^)^(49)^; the remaining two studies reported null findings^(42,66)^. The Alternate Mediterranean Diet Score and the Diet Quality Score were significantly inversely related to VAT adjusted for body size with respective values for extreme quantiles of 163 *v*. 173 cm^2(51)^, 3·8 *v*. 4·5 l^(46)^ and 460·5 *v*. 523·6 cm^2^/m^(56)^. Two Dietary Approaches to Stop Hypertension studies also showed significant inverse associations with VAT^(38,51)^. Regarding plant-based diet indices, adjusted for body size^(21)^, no association of VAT with plant-based diet index or unhealthy plant-based diet index but a –4·9 % significant lower VAT was seen. A higher E-DII was associated with higher VAT with^(39,50)^ and without^(54)^ adjustment for body size. While VAT was 16·9 cm^2^ higher per 1 unit E-DII score without body-size adjustment^(54)^, it differed only by 4·6 cm^2^ after body-size adjustment^(50)^, but the comparability of results from two different studies is limited. In three UPF investigations, one showed no association^(57)^, but the index was significantly associated with VAT per 1 % of total energy intake from UPF in a body-size-adjusted study^(41)^ and in a non-adjusted one^(48)^. Higher levels of VAT with higher levels of fast-food intake were detected with^(55)^ and without adjusting for body size^(55)^.

**Table 5.** Relation of *a priori* diet quality patterns with visceral adipose tissue (VAT) – part 2

Author Year	DQP*	Baseline VAT†	VAT-effect measures by DQP‡	*P*	Body size adjusted	Association with VAT§
De Amicis 2020^(35)^	MED	5·3 ± 2·7 cm	C: 0·01 (–0·08, 0·11) cm	≥0·05	Y	→
Bellissimo 2020^(38)^	MED	0·88 kg	C: −0·06 ± 0·02 (±se) kg	0·003	N	↓
Bertoli 2015^(27)^	MED	4·6 (3·2–6·5) mm	C: −0·05 (–0·08, −0·01) cm^3^	<0·05	N	↓
Flor-Alemany 2020^(66)^	MED	647 ± 295 g	r = −0·11	≥0·05	N	→
Galmes-Panades 2019^(42)^	MED	2·29 ± 0·89 kg	C: −7·16 (–23·7, 9·34) g	0·40	N	→
Hennein 2019^(28)^	MED	Q1:1735 cm^3^ Q4:1744 cm^3^	Q1: 715 (646, 784) cm^3^ Q4: 595 (533, 657) cm^3^ C: −50 (–86, −14) cm^3^	0·007	Y	↓
Holthaus 2023^(43)^	MED	608 ± 324 g	C: −0·19 g	<0·01	N	↓
	DASH	608 ± 324 g	C: −0·22 g	<0·01	N	↓
Jennings 2024^(46)^	aMED	4·1 ± 2·2 L	T1: 4·5 (4·2,4·7) l T3: 3·8 (3·5,4·1) L	<0·01	Y	↓
Lopes 2023^(49)^	MED	142 ± 238 cm^3^	Low: 267 ± 334 cm^3^ High: 106 ± 155 cm^3^	0·005	N	↓
Maskarinec 2017^(51)^	aMED	168 ± 84 cm^2^	T1: 173 (169, 178) cm^2^ T3: 163 (159, 168) cm^2^	NA	Y	↓
	DASH	168 ± 84 cm^2^	T1: 179 (174, 183) cm^2^ T3: 160 (155, 165) cm^2^	NA	Y	↓
Bellissimo 2020^(38)^	DASH	0·88 kg	C: −0·18 ± 0·04 kg (±se)	<0·001	N	↓
Shah 2016^(56)^	Diet Quality Score	NA	Q1: 524 (499, 550) cm^2^/m Q4: 461 (440, 482) cm^2^/m C: −0·009 cm^2^/m	<0·01	Y	↓
Ratjen 2020^(21)^	PDI	3·8 (2·4–5·1) dm^3^	T1: Ref T3: −3·0 (–8·6, 4·1) % C: −2·0 (–6·8, 2·0) %	NA	Y	→
	hPDI	3·8 (2·4–5·1) dm^3^	T1: Ref T3: −5·8 (–12·2, 0·2) % C: −4·9 (–8·6, −2·0) %	NA	Y	↓
	uPDI	3·8 (2·4–5·1) dm^3^	T1: Ref T3: 5·1 (–1·0, 11·6) % C: 3·0 (–1·0, 7·3) %	NA	Y	→
Corrêa 2022^(39)^	E-DII	T1–T3: 412, 449, 1026 g	T1: Ref T2: −614 (-492, 385) g T3: 594 (168, 1019) g	0·13	Y	↑
Lozano 2022^(50)^	E-DII	T1–T3: 142, 168, 192 cm^2^	C: 4·61 (2·95, 6·27) cm^2^	NA	Y	↑
Mtintsilana 2019^(54)^	E-DII	165 ± 62 cm^2^	C: 16·9 (8·3, 25·5) cm^2^	<0·0001	N	↑
Fridén 2022^(41)^	UPF	3·2 (1·7–4·5) L	LnVAT: 0·01 (0·002, 0·02)	0·02	Y	↑
Liu 2023^(48)^	UPF	NA	QT1: 97 (93–100) QT5: 106 (102–110)	<0·001	N	↑
Shim 2023^(57)^	UPF	0·9 ± 0·6 kg	0·025 (–0·018 to 0·068)	NA	N	→
Odedaard 2022^(55)^	Fast-food intake	NA	0–1×/mo: 99 (91, 105) cm^3^ ≥3×/wk: 146 (140, 151) cm^3^	<0·0001	N	↑

* aMED, Alternate Mediterranean Diet Score; DASH, Dietary Approach to Stop Hypertension; DQP, Diet Quality Pattern; E-DII, Energy-Adjusted Dietary Inflammatory Index; hPDI, healthy PDI; MED, Mediterranean Diet Score; PDI, plant-based diet index; UPF, ultra-processed food; uPDI, unhealthy PDI.

† Mean ± sd or median (IQR).

‡ C, continuous score, values reported as ß (95 % CI) unless specified; T, tertile; Q, quartile; QT, quintile; *r*, Pearson correlation coefficient.

§ ↑, significant positive association; ↓, significant inverse association; →, no association.

For the twenty-seven *a posteriori* DQP identified in nine reports (Table 6), six patterns were positively associated with VAT, and four patterns were inversely associated with VAT, whereas seventeen patterns indicated no relation to VAT. Generally, a higher score reflects a stronger adherence to the pattern indicated by the name of the pattern. Significant positive associations were seen for the following dietary patterns: a partial least squares regression-2 pattern high in nutrients found in meat and eggs, fish and beer and low in nutrients found in plants and dairy products in a German population^(60)^, the southern US dietary pattern in African Americans^(26)^, an animal-driven nutrient pattern in South African women^(62,63)^, a retinol and vitamin B_12_-driven nutrient pattern in South Africans^(64)^, a sweet-fast pattern in Chinese men^(65)^ and a *in natura*/minimally processed *v*. processed/ultra-processed pattern among Brazilian men^(58)^. Inverse associations with VAT were identified for the following dietary patterns: a reduced-rank-regression-derived dietary pattern with high intakes of fruits and vegetables, which was linked to variation of selenoprotein P concentrations, in a German population^(59)^, the healthy Japanese dietary pattern in men but not women^(61)^, the vegetable-fruits pattern among Chinese individuals^(65)^ and a principal component analysis-8 characterised by key nutrients in skimmed milk in a Germany study^(60)^. Otherwise, null associations were seen for the remaining patterns based on foods^(26,58,64)^ or nutrients^(62,63)^ in different geographic locations.

**Table 6.** Relation of *a posteriori* diet quality patterns with visceral adipose tissue

Author, Year	DQP*	Baseline VAT†	Effect measures for VAT by DQP‡	*P*	Body size adjusted	Association with VAT§
De Paula Mancilha 2022^(58)^	Natural/minimally (F)	NA	C: 0·15 ± 0·02 kg (ß ± s d)		Y	→
	Ultra-processed (F)	NA	C: 0·11 ± 0·01 kg (ß ± s d)	0·25	Y	→
	Natural/minimally (M)	NA	C: 0·14 ± 0·01 kg (ß ± s d)		Y	↑
	Ultra-processed (M)	NA	C: 0·18 ± 0·02 kg (ß ± s d)	0·02	Y	↑
Di Giuseppe 2019^(59)^	Dietary pattern score	3·9 (2·4–5·2) dm^3^	C: −0·12 (–0·19; −0·05) dm^3^		N	↓
Fischer 2015^(60)^	PCA-1	4·1 ± 2·1 dm^3^	C: 0·01 (–0·05, 0·06) dm^3^	0·8	Y	→
	PCA-2	4·1 ± 2·1 dm^3^	C: 0·03 (–0·02, 0·09) dm^3^	0·2	Y	→
	PCA-3	4·1 ± 2·1 dm^3^	C: −0·03 (–0·08, 0·01) dm^3^	0·1	Y	→
	PCA-4	4·1 ± 2·1 dm^3^	C: −0·02 (–0·07, 0·03) dm^3^	0·4	Y	→
	PCA-5	4·1 ± 2·1 dm^3^	C: −0·02 (–0·07, 0·03) dm^3^	0·4	Y	→
	PCA-6	4·1 ± 2·1 dm^3^	C: −0·03 (–0·08, 0·02) dm^3^	0·2	Y	→
	PCA-7	4·1 ± 2·1 dm^3^	C: <0·001 (–0·05, 0·05) dm^3^	0·99	Y	→
	PCA-8	4·1 ± 2·1 dm^3^	C: −0·06 (–0·10, −0·01) dm^3^	0·01	Y	↓
	PCA-9	4·1 ± 2·1 dm^3^	C: 0·02 (–0·03, 0·07) dm^3^	0·40	Y	→
	PLS-1	4·1 ± 2·1 dm^3^	C: −0·02 (–0·07, 0·03) dm^3^	0·40	Y	→
	PLS-2	4·1 ± 2·1 dm^3^	C: 0·06 (0·01, 0·11) dm^3^	0·02	Y	↑
Ito 2019^(61)^	Healthy Japanese dietary pattern F	T1: 50·9 ± 28·3 cm^2^ T3: 46·2 ± 22·5 cm^2^	T1: 51·1 (44·4, 57·9) cm^2^ T3: 45·5 (38·2, 52·7) cm^2^	0·27	N	→
	Healthy Japanese dietary pattern M	T1: 97·7 ± 40·2 cm^2^ T3: 76·9 ± 37·8 cm^2^	T1: 94·0 (85·6, 102·4) cm^2^ T3: 80·4 (72·5, 88·4) cm^2^	0·03	N	↓
	Seafood and alcohol dietary pattern F	T1: 54·4 ± 27·6 cm^2^ T3: 53·7 ± 33·8 cm^2^	T1: 56·7 (49·2, 64·3) cm^2^ T3: 51·3 (44·0, 58·7) cm^2^	0·34	N	→
	Seafood and alcohol dietary pattern M	T1: 84·4 ± 41·5 cm^2^ T3: 90·0 ± 42·7 cm^2^	T1: 86·5 (78·0, 95·0) cm^2^ T3: 89·3 (81·2, 97·4) cm^2^	0·66	N	→
Liu 2013^(26)^	Fast-food US dietary pattern	T1: 691 ± 17 cm^3^ T3: 731 ± 19 cm^3^	OR: T1:1 T3: 1·52 (0·8, 2·3)	0·14	N	→
	Prudent US dietary pattern	T1: 714 ± 20 cm^3^ T3: 721 ± 18 cm^3^	OR: T1:1 T3: 0·91 (0·6, 1·3)	0·61	N	→
	Southern US dietary pattern	T1: 681 ± 18 cm^3^ T3: 764 ± 18 cm^3^	OR: T1:1 T3: 1·80 (1·1, 3·0)	0·02	N	↑
Makura-Kank- wende 2020^(62)^	Animal driven	100 (74–128) cm^2^	C: 9·88 (5·13, 14·63) cm^2^	<0·001	N	↑
	Plant driven	100 (74–128) cm^2^	C: 2·06 (–3·12, 7·23) cm^2^	0·43	N	→
	Vit C, sugar, potassium	100 (74–128) cm^2^	C: −1·09 (–4·97, 2·80) cm^2^	0·58	N	→
Makura-Kank-wende 2022^(63)^	Animal driven	108 (70–137) cm^2^	C: 5·79 (0·01, 11·57) cm^2^	<0·05	Y	↑
	Plant driven	108 (70–137) cm^2^	C: −4·04 cm^2^	≥0·1	Y	→
	Vit C, sugar, potassium	108 (70–137) cm^2^	C: 0·01 cm^2^	≥0·1	Y	→
Ratshikombo 2021^(64)^	Animal driven	M: 87·4 ± 46·0 cm^2^ F: 104·1 ± 44·3 cm^2^	C: 1·62 (–0·90, 3·94) cm^2^	0·22	Y	→
	Plant driven	M: 87·4 ± 46·0 cm^2^ F: 104·1 ± 44·3 cm^2^	C: 1·12 (–1·28, 3·53) cm^2^	0·36	Y	→
	Retinol and vitamin B_12_ driven	M: 87·4 ± 46·0 cm^2^ F: 104·1 ± 44·3 cm^2^	C: 4·15 (1·86, 6·44) cm^2^	<0·001	Y	↑
	Vit C, sugar, potassium	M: 87·4 ± 46·0 cm^2^ F: 104·1 ± 44·3 cm^2^	C: 0·79 (–1·63, 3·21) cm^2^	0·52	Y	→
Yin 2020^(65)^	Rice-meat pattern	F: 182 (142–224) cm^2^ M: 124 (100–155) cm^2^	r = 0·035	0·40	N	→
			r = −0·007	0·89	N	→
	Seafood-eggs	F: 182 (142–224) cm^2^ M: 124 (100–155) cm^2^	r = 0·061	0·15	N	→
			r = 0·032	0·53	N	→
	Sweet-fast	F: 182 (142–224) cm^2^ M: 124 (100–155) cm^2^	r = 0·012	0·78	N	→
			r = 0·20	<0·001*	N	↑
	Vegetable-fruits	F: 182 (142–224) cm^2^ M: 124 (100–155) cm^2^	r = −0·099	0·02	N	↓
			r = −0·111	0·03	N	↓

* PCA, principal component analysis; PLS, partial least squares regression.

† Reported as mean ± sd or median (IQR).

‡ Unless specified, values reported as ß (95 % CI) or for tertiles, values are lowest (T1) and highest tertile (T3); C: continuous; r: Pearson coefficient.

§ ↑, significant positive association; ↓, significant inverse association; →, no association.

A serious risk of bias according to ROBINS-I was detected in twenty-eight reports (online Supplementary Table 3). Selection bias was deemed as a serious risk in thirteen studies due to the convenience sampling of participants. Missing data were rated as a serious threat in thirteen studies as less than half of the original participants were analysed. Nevertheless, it is important to note that in cohort studies, due to pragmatic reasons, only a subgroup can undergo imaging. The outcome assessment was judged as having a low risk of bias for all included studies, given that only studies with valid imaging techniques were included. Exposure assessment was considered as a moderate risk of bias for most studies; only ten studies using short-item questionnaires or 24-d recalls were rated as serious. The risk of bias due to selective reporting was deemed to be serious in six studies because the association between DQP and VAT was not the primary objective of the study, whereas this risk appeared moderate for the rest.

## Discussion

The present systematic review provides a detailed evaluation of *a priori* and *a posteriori* DQP assessed in relation to VAT across different populations. The thirty-five studies revealed inverse associations between DQP and VAT, which were stronger for *a priori* than *a posteriori* DQP, younger *v*. older populations, studies in the USA as compared with elsewhere, multi-ethnic studies *v*. one group only and investigations with moderate *v*. serious risk of bias. No obvious differences were detected by dietary assessment method, imaging method, year of publication and body-size adjustment.

The majority of *a priori* indices representing dietary guidelines, that is, HEI, Alternate HEI, Dutch Dietary Guidelines Index and Dietary Guidelines for Americans Adherence Index, showed significant inverse associations with VAT. Apart from a few exceptions, indices based on specific diets, that is, Dietary Approaches to Stop Hypertension, healthy plant-based diet index and MED, were also inversely associated with VAT, while E-DII, UPF and fast-food intake were positively associated with higher VAT. In contrast, only about one in three *a posteriori* patterns was significantly associated with VAT, including a healthy Japanese dietary pattern, a vegetable-fruits pattern and the skimmed milk nutrient pattern and, positively, the southern US dietary pattern, the animal-driven nutrient pattern, the retinol and vitamin B_12_ pattern and the sweet-fast pattern.

The weaker associations for *a posteriori* patterns may be due to their data-driven nature, involving a subjective selection of specific food items and decision-making based on correlations among foods or nutrients consumed without a clear underlying hypothesis about the relation of diet to health^(23)^ as compared with *a priori* DQP developed as guidelines based on the scientific literature^(11)^. As higher scores of several *a priori* DQP have been linked to positive health effects^(24)^, it is possible that the beneficial health effects of high diet quality can be partly attributed to their potential lowering effect on VAT. Several *a posteriori* DQP, for example, the ‘prudent’ or ‘healthy pattern’, share an emphasis on vegetables and fruits with the *a priori* indices^(22)^, and higher scores in these patterns appear to be protective against different diseases^(67)^. DQP that focus on nutrient density and/or low-energy foods may be effective through the same pathway^(68)^. Higher scores of DQP describing a pro-inflammatory diet, that is, UPF, fast food and E-DII, were positively associated with VAT probably as a result of high total energy, saturated fat, cholesterol and sugar content^(69)^, which is linked to an increased chronic disease risk^(70)^. Some *a posteriori* DQP reflect a diet often described as ‘western’ or ‘unhealthy’ diet pattern^(22)^ and linked to chronic inflammation^(6)^, which may mediate the association between poor quality diet and VAT^(71)^. Contrary to expectation, the lack of adjustment for body size^(1)^ did not affect the ability of detecting associations between DQP and VAT, but the discrepant methods limited our ability to evaluate differences in the magnitude of effect measures, a necessary tool to identify the influence of DQP on VAT independent of BMI^(1)^. However, individual studies support the hypothesis that the association weakens after adjustment for body size; for example, in a Dutch investigation, a higher DQP score showed lower VAT levels of –2·3 *v*. –3·2 cm^2^ with and without including body size into the model^(20)^.

Several limitations of the present review need to be considered, notably the relatively serious risk of bias inherent to observational studies^(37)^. Despite the relatively low bias for the outcome measure and the moderate bias for selective reporting and confounding, the high proportions of selection bias and overall serious bias introduce unavoidable uncertainty about the accuracy of the findings^(37)^. As to design features, the different body fat assessment methods are of concern as they result in units that are difficult to compare^(2)^. However, the validity of DXA^(32–34)^ as well as ultrasound^(36)^ in relation to MRI and CT has been demonstrated repeatedly^(32)^, and the reliance on state-of-the-art methods for assessing adiposity represents a considerable strength^(72)^. The heterogeneity of statistical approaches, that is, continuous or categorical analysis, with or without body-size adjustment, made it impossible to estimate a quantitative summary of the findings as is typically done in meta-analyses. The challenges of different dietary assessments always need to be considered^(73)^. While most studies used FFQ covering the previous month or year, 24-h recalls or dietary records are even more prone to measurement error due to the short time covered, but under- and over-reporting as a result of self-report is common across methods. By limiting the studies to disease-free adolescents and adults, English language and full-text availability, some findings may have been missed and do not apply to chronically diseased individuals and children. Comparability of studies was limited by differences in dietary behaviour and intake across geographic regions and ethnic composition. Thus, the DQP may not capture diet quality to the same degree across populations; for example, the MED score was initially developed for the Greek population^(74)^ and may not capture regular dietary intake as well among non-European ethnic groups^(51)^. This may explain the discrepant findings for the MED (Table 5). Also, *a priori* DQP are restricted by the current level of knowledge regarding the relation between diet and disease^(12)^. As recommendations can be met by making different decisions, for example, choosing raw *v*. processed fruits or using distinctive cooking methods, they may experience different health effects despite similar scores. As all findings were based on cross-sectional investigations, the causality of the relations cannot be ascertained, and the quality of included studies was judged to be at moderate or serious risk of bias (online Supplementary Table 3)^(75)^. Large cohorts, such as the German National Cohort (NAKO) and the UK Biobank, are now collecting imaging data for large numbers of individuals and will be able to conduct prospective analyses in the future.

### Conclusions

This comprehensive review of thirty-five cross-sectional studies of diet quality in relation to VAT shows stronger evidence for an inverse association in studies with *a priori* than *a posteriori* DQP, possibly due to their development based on evidence-based food-disease relations^(11)^. Yet, the heterogeneity across studies limits the ability to compute a quantitative summary measure of association. As the majority of DQP reflect high consumption of plant-based foods and low intake of animal products, the specific choice of DQP for public health interventions may be less important than the overall goal of achieving high diet quality, which can be achieved in different ways such as various combinations of food that are locally available and culturally acceptable. This review may serve as a basis for analyses in large prospective cohorts and for future interventions targeting VAT reduction at the population level.

## Supporting information

Thimm et al. supplementary material Thimm et al. supplementary material

## Supplementary material

For supplementary material/s referred to in this article, please visit https://doi.org/10.1017/S000711452400179X.
