# Supplementary material for: A systematic review of observational studies on the association between diet quality patterns and visceral adipose tissue
Source: Br J Nutr. 2024 Nov 12;132(11):1530–41. doi: 10.1017/S000711452400179X (PMC11660310; doi:10.1017/S000711452400179X)
Supplement: Thimm et al. supplementary material [file S000711452400179Xsup001.docx]

**Supplemental Table 1:** Search string for PubMed and EMBASE (simplified)

| Search component | Search term ("key words") three search subterms from PubMed |
| --- | --- |
| "Exposure" | "Healthy Eating Index" OR "HEI" OR "HEI-2010" OR "Alternative Healthy Eating Index" OR "AHEI-2010" OR "alternate Mediterranean Diet score" OR "mediterranean diet" OR "aMED" OR "Dietary Approaches to Stop Hypertension" OR "DASH" OR "Mediterranean Diet Score" OR "MDS" OR "Diet Quality Index" OR "DQI" OR "Healthy Diet Indicator" OR "HDI"  **OR**  (diet* OR eating OR food)  **AND** (quality OR index OR indices OR indexes OR score OR scores OR behaviour OR pattern OR patterns OR factors OR habits) |
| "Outcome" | "body fat distribution"  **OR**  visceral **AND** (adiposity OR "adipose tissue" OR obesity OR fat OR "body fat" OR "abdominal tissue" OR "abdominal fat") |
| "Measurement of the outcome" | "imaging" OR "magnetic resonance imaging" OR "MRI" OR "dual‑energy X‑ray‑absorptiometry" OR "DXA" OR "DEXA" OR "computed tomography" OR "CT" OR ultrasound OR ultrasonography OR "VAAT" OR "VAT" OR "VFA" |

**Supplemental Table 2:** Description and decision criteria for domains in ROBINS-I (adapted from Pinart 2021)

| Domain | Explanation | Judgements |
| --- | --- | --- |
| Bias due to confounding | Is there potential for confounding of the effect of exposure in this study?  Did the authors use an appropriate analysis method that controlled for all the important confounding domains (age + sex + body-size)  Notes: Confounding is expected in all observational studies. | Low risk of bias: No confounding expected.  Moderate risk of bias: Confounding expected: Age, sex and body-size were appropriately measured or controlled for in a multivariable-adjusted analysis.  Serious risk of bias: At least one known important confounding factor was not measured or appropriately controlled for.  No information: No information on which confounder have been controlled for. |
| Bias due to selection of participants | Was selection of participants into the study representative of the average in the target population (all subjects or random sampling)?  Were adjustment techniques used that are likely to correct for the presence of selection biases?  Notes: In observational studies, it is unlikely that post-exposure variables influenced selection of participants into the study. Exclusion of participants may be mostly based on missing data, which will be considered in the domain referring to missing data (see below). | Low risk of bias: All participants were selected from the population using random sampling methods and the response rate is not lower than 80%.  Moderate risk of bias: Selection into the study was somewhat representative of the average in the target population (non-random sampling).  Serious risk of bias: Participants were selected by means of a convenient sample (no sampling strategy performed).  No information: No information is reported about selection of participants into the study. |
| Bias due to exposure assessment | Was the exposure status clearly defined and adequately assessed?  Notes: Bias introduced by either differential or non-differential misclassification of exposure status.  Non-differential misclassification is unrelated to the outcome and will usually bias the estimated effect of exposure towards the null. Differential misclassification occurs when misclassification of exposure status is related to the outcome or the risk of the outcome, and is likely to lead to bias. | Low risk of bias: Exposure status was well defined; and no measurement error is expected in its assessment.  Moderate risk of bias: Exposure status was well defined; and exposure was measured subjectively by FFQ with high number of items.  Serious risk of bias: Exposure status was not well defined or exposure was measured subjectively by FFQ with low number of items or 3-day recalls.  No information: Exposure status was not defined or no information about the exposure assessment was reported. |
| Bias due to missing data | Were participants excluded due to missing data on exposure status or on other variables needed for the analysis (e.g., outcome)?  Notes: Missing data on exposure variables and other variables are expected to be missing at random and not related to exposure or outcome. | Low risk of bias: Data on exposure and outcome variables were reasonably complete (<10% missing data).  Moderate risk of bias: There is a proportion of missing exposure or outcome data of >10% and <50% between initially eligible sample vs. finally analysed sample.  Serious risk of bias: High proportions (>50%) of missing exposure or outcome data between initially eligible sample vs. finally analysed sample.  No information: No information was reported about missing data or the potential for data to be missing. |
| Bias due to measurement of the outcome | Could the outcome measure have been influenced by knowledge of the exposure status?  Was any systematic error in measurement of the outcome related to exposure status?  Notes: In observational studies, it is not expected that outcome assessors were aware of exposure status of the participants. | Low risk of bias: The outcome measure was unlikely to be influenced by knowledge of the exposure status of study participants; and any error in measuring the outcome was unrelated to exposure status (i.e., objective measures such as imaging methods).  Moderate risk of bias: Any error in measuring the outcome was only minimally related to exposure status.  Serious risk of bias: Error in measuring the outcome was related to exposure status.  No information: No information is reported about the methods of outcome assessment. |
| Bias due to selective reporting of the results | Is the reported effect estimate likely to be selected from multiple analyses of exposure-outcome relationship? Is the reported effect estimate likely to be selected from different subgroups?  Notes: In observational studies, it is unusual to publish an *a priori* analysis plan or protocol. | Low risk of bias: There is clear evidence that all reported results correspond to all intended outcomes, analyses and sub-cohorts.  Moderate risk of bias: The outcome measurements and analyses are clearly defined; and there is no indication of selection of the reported analysis from among multiple analyses; and there is no indication of selection of the cohort or subgroups for analysis and reporting on basis of the results (e.g., estimates not shown for all analyses).  Serious risk of bias: There is a high risk of selective reporting from among multiple analyses (e.g., association between diet quality pattern & VAT was not their primary objective); or the cohort or subgroup is selected from a larger study for analysis and appears to be reported based on the results.  No information: There is too little information to make a judgement. |
| Overall judgement | Low risk of bias | The study is judged to be at low risk of bias for all domains. |
|  | Moderate risk of bias | The study is judged to be at low or moderate risk of bias for all domains. |
|  | Serious risk of bias | The study is judged to be at serious risk of bias in at least one domain, but not at critical risk in any domain. |

**Supplemental Table 3:** Critical appraisal of the included studies using ROBINS-I tool^1^

| **Author, Year** | **Body-size adjustment** | **Confounding** | **Selection bias** | **Exposure assessment^2^** | **Missing**  **Data^3^** | **Outcome assessment** | **Selective**  **reporting** | **Overall**  **judgement** |
| --- | --- | --- | --- | --- | --- | --- | --- | --- |
| Bellissimo, 2020^(50)^ | Ø | S | M | M (FFQ) | L (693/739) | L | S (not primary) | **S** |
| Bertoli, 2015^(27)^ | Ø | S | S (no sampling method) | S (14-item qx) | No information | L | M | **S** |
| Corrêa, 2022^(38)^ | Relative fat mass | M | S (campus) | S (24h) | No information | L | M | **S** |
| De Amicis, 2020^(35)^ | BMI | M | S (no sampling method) | S (14-item qx) | M (416/476) | L | S (not primary) | **S** |
| De Paula Mancilha, 2022^(39)^ | Birth weight | M | S (school) | M (FFQ) | M (238/450) | L | S (not primary) | **S** |
| Di Giuseppe, 2019^(58)^ | Ø | S | M | M (FFQ) | S (553/1316) | L | S (not primary) | **S** |
| Fischer, 2015^(59)^ | BMI/Height | M | M | M (FFQ) | S (583/1316) | L | S (not primary) | **S** |
| Flor-Alemany, 2020^(61)^ | Ø | S | S (trial) | M (FFQ) | M (176/198) | L | M | **S** |
| Fridén, 2022^(63)^ | BMI | M | M | M (FFQ) | M (286/502) | L | M | **M** |
| Galmes-Panades, 2019^(62)^ | Height | M | S (trial) | M (FFQ) | S (1231/6874) | L | M | **S** |
| Hennein, 2019^(28)^ | BMI | M | M | M (FFQ) | S (1677/>6000) | L | M | **S** |
| Holthaus, 2023^(49)^ | Ø | S | S (newsletter) | M (FFQ) | No information | L | M | **S** |
| Hu, 2023^(45)^ | Body composition | M | S (convenience) | S (24h) | S (192/342) | L | M | **S** |
| Isanejad, 2023^(48)^ | Height | M | M | M (FFQ) | S (3017/5155) | L | M | **S** |
| Jennings, 2024^(60)^ | Height | M | M | M (FFQ) | S (620/1316) | L | M | **M** |
| Ito, 2019^(64)^ | Ø | S | S (convenience) | S (BDHQ) | S (421/1016) | L | M | **S** |
| Landry, 2019^(40)^ | BMI percentile | M | S (campus) | S (24h) | L (92/100) | L | M | **S** |
| Lopes, 2023^(47)^ | Ø | S | S (campus) | S (14-item qx) | No information | L | M | **S** |
| Liu, 2013^(26)^ | Ø | S | M | S (24h) | S (1775/5301) | L | M | **S** |
| Liu, 2023^(44)^ | Ø | S | M | S (24h) | S (9640/13044) | L | M | **S** |
| Lozano, 2022^(51)^ | Total body fat | M | M | M (FFQ) | M (1655/1861) | L | M | **M** |
| Makura-Kankwende, 2020^(42)^ | Ø | S | M | M (FFQ) | M (498/702) | L | M | **S** |
| Makura-Kankwende, 2022^(42)^ | Fat mass index | M | M | M (FFQ) | S (132/527) | L | M | **S** |
| Maskarinec, 2017^(52)^ | Total body fat | M | M | M (FFQ) | L (1765/1861) | L | M | **M** |
| Maskarinec, 2020^(53)^ | Total body fat | M | M | M (FFQ) | L (1765/1861) | L | M | **M** |
| Molenaar, 2009^(54)^ | Ø | S | M | M (FFQ) | S (1926/5209) | L | M | **S** |
| Mtintsilana, 2019^(57)^ | Ø | S | M | M (FFQ) | No information | L | M | **S** |
| Odegaard, 2022^(46)^ | Ø | S | M | S (24h) | M (3156/5115) | L | M | **S** |
| Panizza, 2020^(29)^ | Total body fat | M | S (convenience) | M (FFQ) | M (468/540) | L | M | **S** |
| Ratjen, 2020^(21)^ | BMI | M | M | M (FFQ) | M (578/952) | L | M | **M** |
| Ratshikombo, 2021^(56)^ | Total body fat | M | L (random) | M (FFQ) | S (760/2035) | L | M | **S** |
| Shah, 2016^(55)^ | Weight | M | M | M (FFQ) | M (5076/6814) | L | M | **M** |
| Shim, 2023^(65)^ | Ø | S | M | M (FFQ) | S (2303/4431) | L | S (missing VAT) | **S** |
| van Eekelen,2020^(20)^ | Total body fat | M | S (oversampling) | M (FFQ) | M (3912/6671) | L | M | **S** |
| Yin, 2020^(41)^ | Ø | S | NI | M (FFQ) | No information | L | M | **S** |

^1^L: low risk of bias; M: moderate risk of bias; S: serious risk of bias

^2^(24h): 24-hour recall or 3-day record; BDHQ: brief-type self-administered diet history questionnaire; FFQ: food frequency questionnaire; qx: questionnaire

^3^analysed sample size/eligible sample size; NI: no information
